# Toward a Comparison of Classical and New Privacy Mechanism

**DOI:** 10.3390/e23040467

**Published:** 2021-04-15

**Authors:** Daniel Heredia-Ductram, Miguel Nunez-del-Prado, Hugo Alatrista-Salas

**Affiliations:** Engineering Department, Universidad del Pacífico, Lima 15076, Peru; d.herediaductram@alum.up.edu.pe (D.H.-D.); h.alatristas@up.edu.pe (H.A.-S.)

**Keywords:** privacy, statistical disclosure control, generative adversary networks, differential privacy, knowledge distillation

## Abstract

In the last decades, the development of interconnectivity, pervasive systems, citizen sensors, and Big Data technologies allowed us to gather many data from different sources worldwide. This phenomenon has raised privacy concerns around the globe, compelling states to enforce data protection laws. In parallel, privacy-enhancing techniques have emerged to meet regulation requirements allowing companies and researchers to exploit individual data in a privacy-aware way. Thus, data curators need to find the most suitable algorithms to meet a required trade-off between utility and privacy. This crucial task could take a lot of time since there is a lack of benchmarks on privacy techniques. To fill this gap, we compare classical approaches of privacy techniques like Statistical Disclosure Control and Differential Privacy techniques to more recent techniques such as Generative Adversarial Networks and Machine Learning Copies using an entire commercial database in the current effort. The obtained results allow us to show the evolution of privacy techniques and depict new uses of the privacy-aware Machine Learning techniques.

## 1. Introduction

Nowadays, we live in an interconnected world where much data is generated from sensors, social networks, internet activity, etc., which can be found in various data repositories. This data may contain sensitive information that can be revealed when it is they are analyzed. To address this problem, many data sanitization mechanisms were proposed to provide some privacy guarantees. Conversely, from an organizational perspective, data also hide patterns that help in the decision-making process. In this context, sanitizing algorithm’s challenge is twofold: how data could be shared containing useful information but respectful of privacy.

Various algorithms are racing against each other to provide the highest privacy without penalizing data utility for mining tasks. Therefore, data curators need to test several algorithms to find a suitable solution to satisfy the trade-off between privacy and utility. In the literature, there are few benchmarks comparing privacy algorithm performance. To the best of our knowledge, there is a lack of benchmarks, including recent privacy algorithms based on Deep Learning and Knowledge Distillation. Accordingly, to fill this gap, in the present study, we performed a benchmark between classical mechanisms, such as those based on Statistical Disclosure Control, including filters such as Noise Addition, Microaggregation, and Rank swapping filters. Besides, within this comparison, we added the Differential Privacy through Laplacian and Exponential mechanisms. Finally, two privacy mechanisms based on Deep Learning were also compared: the mechanism based on Generative Adversary Networks and the Machine Learning Copies.

To compare the algorithms cited above, two measures widely used in the literature [1,2,3,4,5,6] were used, namely, Disclosure Risk and Information Loss. The former quantifies the danger of finding the same distribution for the output variable after a prediction task when the input dataset is sanitized. The latter measures the amount of helpful information loss after applying a sanitization algorithm.

Concerning our results, each sanitization mechanism was tuned to find the best hyperparameters to meet a trade-off between the Information Loss and the Disclosure Risk. Our findings showed the best values of Disclosure Risk measure for Noise Addition, Rank Swapping, and Machine Learning copies. Conversely, Machine Learning copies, Noise addition, and Rank swapping mechanisms have the smallest Information Loss values.

The following list summarizes the major contributions of our paper:

1. Seven sanitization filters were formally defined and compared on a real datasets.
2. Hyperparameters fine-tuning were performed for each mechanism.
3. Two well-known measures were used to select the best mechanism.

The remaining of this paper is organized as follows. Section 2 presents the state-of-the-art, while Section 3 introduces some basic concepts and methods respectively. Section 4 and Section 5 describe the results and the discussion of our proposal. Finally, Section 6 concludes the paper and presents new research avenues.

## 2. Literature Review

This section discusses the most relevant documents in the literature concerning privacy algorithms from two points of view.

### 2.1. Privacy Algorithms Definitions

This subsection describes several privacy algorithm. Accordingly, the first privacy method to be describe is the Statistical Disclosure Control (SDC). For instance, Pietrzak [6] apply SDC filters on data from labor force surveys, which is applied in subsequent forecasting tasks-such as regressions-to estimate the unemployment rate. The main conclusion is the influence of the SDC filter hyperparameters selection on the impact of data utility and confidentiality. Another work proposed by Andrés et al. [7] propose a geo-Indistinguishability mechanism for Location-Based Services (LBS) combining Laplacian Differential Privacy and *k*-anonymity.

In the same spirit, Parra-Arnau et al. [8] introduce a new Microaggregation-based filter called Moment-Microaggregation. This new technique aims to substitute the original dataset *X* to a new dataset $X^{\prime }$, trying to keep utility for prediction tasks. The principle is to group data points and replace them with some statistical values like the mean. Later, from the $X^{\prime }$ dataset, the authors apply a Differential Privacy mechanism [9] to obtain a new dataset $X^{\prime \prime }$. Finally, the latter dataset provides the best privacy guarantees and utility of the sanitized information. Anther work presented by Nin et al. [10] suggest the Rank swapping algorithm to reduce Disclosure Risk, a well-known metric used to evaluate privacy algorithms’ performance. The main idea is to change each variable’s values with other records within a restricted range (a window). This new value is used as a hyperparameter of the algorithm. As a result, the authors obtain a significant reduction in Disclosure Risk compared to other methods. Regarding the data privacy techniques application on industrial sectors, Altman et al. [11] use different privacy techniques within traditional business processes, incorporating several layers of protection: explicit consent, systematic review, Statistical Disclosure Control (SDC), procedural controls, among others. In the same spirit, [12] compares some of the most used privacy methods in companies, namely *k*-anonymity, *l*-diversity, and randomization. Results show that, although the methods provide a certain privacy guarantee while preserving usefulness for prediction models. The authors also state that new methods must be proposed to deal with certain disadvantages of the privacy methods used, such as time complexity. Finally, the Internet Industry CONSORTIUM [13] concludes that the privacy measures and filters evaluated in the research work, taken in different sectors in recent years (until before 2019), are found based on still traditional and ineffective techniques, as the basic anonymization filter.

Concerning Deep Learning techniques, the training dataset could be reconstructed from the synthetic data [14]. Thus, Xie et al. [15] propose to apply ϵ-Differential Privacy to the training dataset before passing it as the input of the Wasserstein Generative Adversary Networks (WGAN) algorithm. The authors test the influence of the ϵ parameter in the data generation for a classification task. They used the MINIST and Electronic Health Records for the experiments showing that the higher the ϵ the lower the privacy warranty and the higher the classification accuracy. Xu et al. [16] propose the GANObfuscator framework, which uses Differential Privacy Generative Adversary Networks algorithm to built synthetic data from real medical reports. The basic idea is to add noise into the learning process of the WGAN by injecting ϵ bounded random noise, sampled from a normal distribution, in the discriminator updating. The scientists use MINIST, LSUN, and CelebA datasets to generate synthetic data and a classification task to measure data utility. The authors state that the new data show a moderate Disclosure Risk, maintaining the data high utility for subsequent classification tasks. In the same spirit, Triastcyn and Faltings [17] propose a differential private DCGAN by adding Gaussian distribution noise in the discriminator weights to meet Differential Privacy guarantees in the synthetic data by the generator output. As previously mentioned, the author relies on the MINIST and SVHN datasets to generate synthetic datasets for a classification task.

More recently, Machine Learning copies [18] has been used to remove sensitive data. For instance, the work of Unceta, Nin, and Pujol [19] propose a Machine Learning copy using Artificial Neural Networks and Decision Trees to generate synthetic datasets. The idea behind this technique is to train a classifier with an original dataset. Once the classifier is trained, they put aside the original dataset and generate a new input dataset sampling from a Normal or Uniform distributions, respectively. Thus, this new synthetic dataset could be used to train another classifier. Finally, Gao and Zhou [20] propose a framework combining GAN and Knowledge Distillation. The authors use three networks, namely a teacher, a student, and a discriminator. Thus, the teacher is trained with a sensible dataset, and the outputted data from the teacher is used for the student learning. Then, the student acts as a generator, and a Rényi Differential Privacy mechanism is implemented in the output of the discriminator to modify the feedback to the generator (student). Authors measure their proposal’s performance based on a classification task using the MNIST, SVHN, and CIFAR datasets. The results show an accuracy between 78% and 98% for the classification task.

### 2.2. Privacy Algorithms Benchmark

This subsection describes some benchmarks found in the literature. Concerning de-identification techniques comparison, Tomashchuk et al. [21] propose a benchmark of de-identification algorithms, such as aggregation, top/bottom coding, suppression, and shuffling for achieving different *k*-anonimity like privacy guarantees. They measure the algorithm performance using the *Discernibility Metric*, which reflects the equivalence class size, and the *Normalized Average Equivalence Class Size Metric* that measures the data utility change due to aggregation and rounding. Similarly, Prasse, Kohlmaye, and Kuhn [22] compare anonymity algorithms, namely *k*-anonymity, *l*-diversity, *t*-closeness and δ-presence. They use generic search methods such as Incognito Algorithm, Optimal Lattice Anonymization, Flash Algorithm, Depth-First, and Breadth-First to assess anonymity. The authors evaluate the before mentioned algorithms in terms of the number of *transformations that were checked for anonymity*, that measures for the pruning power of the approaches giving an indication of the algorithm performance; the *number of roll-ups* performed. Roll-up is an optimization metric to capture the equivalence classes of a more generalized representation built by merging the equivalence classes; and the execution time of the algorithm. The authors conclude that there is no single solution fitting all needs.

Concerning performance benchmarks, Bertino, Lin, and Jiang [23] propose a benchmark of Additive-Noise-based perturbation, Multiplicative-Noise-based perturbation, *k*-Anonymization, SDC-based, and Cryptography-based privacy-preserving data mining (PPDM) algorithms. To compare the privacy algorithms, they rely on the *privacy level*, which measures how closely the hidden sensitive information can still be estimated; the *hiding failure*, that is the sensitive information fraction not hidden by the privacy technique; the *data quality* after the application of the privacy technique; and the algorithm *complexity*. The authors conclude that none of the evaluated algorithms outperform concerning all the criteria. More recently, Martinez et al. [24] proposes a benchmark of SDC techniques in a streaming context. The authors claim that these techniques are suitable for both business and research sectors. Besides, they found that the Microaggregation filter provides the best results.

In the same spirit, Nunez-del-Prado and Nin [25] study data privacy in streaming context. To achieve this, the authors compare three SDC methods for stream data, namely, Noise addition, Microaggregation, and Differential Privacy. These algorithms were used over a CDR dataset composed of around 56 million events from 266,956 users. The dataset contains four attributes, namely, ID, time-stamp, latitude, and longitude. Concerning the evaluation metrics, the authors use the Sum of Square Errors and the Kullback–Leibler (KL) divergence to measure the Information Loss. Concerning the Disclosure Risk, the authors focus on two possible attacks. On the one hand, the authors use the Dynamic Time Warping adversary model, in which the intruder has access to a part of the original calls, and he wants to link them with their corresponding anonymous data. On the other hand, the authors use the home/work inference, whose goal is to recover a given user’s home or work location from its anonymized records.

Although the bibliographic review shows different privacy methods applied to different domains, one question could be about the most suitable technique to protect a given dataset. Also, there exists a lack of benchmarks comparing classic and more state-of-the-art privacy algorithms. Besides, the metrics they use to compare the algorithms are quite difficult to understand. Thus, a benchmark of privacy methods is required. In this context, several sanitization techniques are compared in this work in terms of Information Loss and Disclosure Risk, keeping in mind that the best methods guarantee data privacy without losing the information utility for subsequent Machine Learning tasks.

## 3. Materials and Methods

In the present section, we introduce the concepts of the Statistical Disclosure Control filters, Differential Privacy, Generative Adversarial Networks, Knowledge Distillation, as well as the Information Loss and Disclosure Risk functions.

### 3.1. Statistical Disclosure Control

The Statistical Disclosure Control (SDC) aims to protect the users’ sensitive information by applying methods called filters while maintaining the data’s statistical significance. It is important to indicate that only disturbing filters have been selected because re-identification is more complex than undisturbed values. Furthermore, the Noise Addition, Microaggregation, and Rank swapping filters have been chosen for their use in the literature [1,24,26].

First, the Noise Addition filter [27] adds uncorrelated noise from a Gaussian distribution to a given variable. This filter takes a noise parameter *a* in the range [0,1]. The *i*-th value of the *x* attribute is denoted as $x_{i}$, while $x_{i}^{\prime }$ indicates its sanitized counterpart. Thus, the obfuscated values are calculated as shown below.

(1) $$x_{i}^{\prime }=x_{i}+a\times \sigma \times c$$

where σ is the standard deviation of the attribute to be obfuscated, and *c* is a Gaussian random variable such that c∼N(0,1).

Second, the Microaggregation filter [28] groups registers into small sets that must have a minimum number of *k* elements. Furthermore, this filter complies with the property of *k*-anonymity. It means that each released register cannot be distinguished from at least k−1 registers belonging to the same dataset. The Microaggregation filter is divided into two steps: partition and aggregation. In the former, registers are placed in various sets based on their similarity containing at least *k* records. These similar sets of registers can be obtained from a clustering algorithm. The latter, the aggregation stage, computes the centroid for each group to replace each group’s elements with their respective centroid value.

Third, the Rank swapping filter [10] transforms a dataset by exchanging the values of confidential variables. First, the values of the target variable are ordered in ascending order. Then, for each ordered value, another ordered value is selected within a range *p*, which is the parameter that indicates the maximum exchange range. A particular value will then be exchanged within the *p* windows.

### 3.2. Differential Privacy

Intuitively Differential Privacy [29] tries to reduce the privacy risk when someone has their data in a dataset to the same risk of not giving data at all. Thus, an algorithm is said to be differential private when the result of a query is hardly affected by the presence or absence of a set of records. Formally, an algorithm *A* is said to be ϵ-differential private if for two datasets $D_{1}$ and $D_{2}$ that differ by at least one record and for all S⊆Range(A):

(2) $$Pr\left[A\left(D_{1}\right)\in S\right]\leq e^{\epsilon }.Pr\left[A\left(D_{2}\right)\in S\right]$$

The larger the value of the ϵ parameter, the weaker the algorithm’s privacy guarantee. Therefore, ϵ usually takes a small value since it represents the probability to have the same output from two datasets, one sanitized and another original [30]. Hence, a small value of ϵ means a little probability of obtaining the same value of the original dataset while using the sanitized dataset (i.e., Disclosure Risk). Later work has added the δ parameter, which is a non-zero additive parameter. This parameter allows ignoring events with a low probability of occurrence. Therefore, an algorithm *A* is (ϵ,δ)-differentially private if for two datasets $D_{1}$ and $D_{2}$ that differ by at least one record and for all S⊆Range(A):

(3) $$Pr\left[A\left(D_{1}\right)\in S\right]\leq e^{\epsilon }.Pr\left[A\left(D_{2}\right)\in S\right]+\delta$$

This technique provides privacy to numeric data using the Laplacian Differential Privacy mechanism [31,32]. Thus, given a *D* dataset, a *M* mechanism (filter) reports the result of a *f* function reaching ϵ-Differential Privacy if M(D)=f(D)+L. Where *L* is a vector of random variables from a Laplace distribution, and f(D) is the Microaggregation filter function. Accordingly, to implement Differential Privacy, the Laplacian or the Exponential mechanism can be used.

On the one hand, the Laplacian mechanism [29] adds random noise to a query’s answers calculated on the available data. Noise is calibrated through a function called sensitivity $S\left(f\right)=\max\{||f\left(D_{1}\right)-f\left(D_{2}\right)|{|}_{1}\}$, which measures the maximum possible change resulting from a query due to the sum or subtraction of a data record. Also, we define Lap(b), which represents a Laplace distribution with scale parameter *b* and location parameter 0. If the value of *b* is increased, the Laplace function curve tends to be a platicurtic shape, allowing higher noise values and, consequently, better privacy guarantees. Therefore, a value is sanitized by the Laplacian mechanism and satisfies the epsilon-Differential Privacy if $San_{f}\left(D\right)=f\left(D\right)+Lap\left(S\left(f\right)/\epsilon \right)$. Where f(D) is a query on the dataset *D* and Lap(S(f)/ϵ) represents the noise extracted from a Laplace distribution with a scale of S(f)/ϵ and location 0.

On the other hand, the Exponential mechanism [33] provides privacy guarantees to queries with non-numerical responses, for which it is not possible to add random noise from any distribution. The intuition is to randomly select an answer to a query from among all the others. Each answer has an assigned probability, which is higher for those answers more similar to the correct answer. Given *R* the range of all possible responses to a query function *f*, and given $u_{f}\left(D,r\right)$ a utility function that measures how good response is r∈R for the query *f* on the dataset *D*, where higher values of $u_{f}$ show more trustworthy answers. In this way, the sensitivity $S(u_{f})$ is defined as the maximum possible change in the utility function $u_{f}$ given the addition or subtraction of a data record.

(4) $$S\left(u_{f}\right)=\max_{Datasets\;D_{1},D_{2},\;\mathrm{and}\;r\in R}\left\{|\right|u_{f}\left(D_{1},r\right)-u_{f}\left(D_{2},r\right){\left|\right|}_{1}\}$$

Given a dataset *D*, a mechanism satisfies ϵ-Differential Privacy if it chooses an answer *r* with probability proportional to $\exp(\frac{\epsilon }{S(u_{f})}u_{f}\left(D,r\right))$. In the present effort, we used the Microaggregation filter in addition to Laplacian and Exponential distribution, respectively, to implement ϵ-differential privacy methods.

### 3.3. Generative Adversary Networks

The Generative Adversary Networks (GAN) [34] comprises both a generative *G* and a discriminatory *D* models. The former captures the distribution of the input dataset. The latter estimates the probability that a sample comes from the real dataset rather than a sample generated by *G*, which is synthetic data. The training procedure for *G* is to maximize the probability that *D* will not be able to discriminate whether the sample comes from the real dataset. Multilayer Neural Perceptron (MLP) can define both models so that the entire system can be trained with the backpropagation algorithm. The following equation defines the cost function:

(5) $$\min_{G}\max_{D}V\left(D,G\right)=E_{x∼p_{data}\left(x\right)}\left[logD\left(x\right)\right]+E_{x∼p_{z}\left(z\right)}\left[log\left(1-D\left(G\left(z\right)\right)\right)\right]$$

The *D* discriminator seeks to maximize the probability that each piece of data entered into the (D(x)) model will be classified correctly. If the data comes from the real distribution or the *G* generator, it will return one or zero, respectively. The generator *G* minimizes the function log(1−D(G(z))). Thus, the idea is to train the generator until the discriminator *D* is unable to differentiate if an example comes from real or synthetic dataset distributions. Hence, the idea is to generate a synthetic dataset $X^{\prime }$ to mimic the original dataset *X*. In this context, the generator’s error to built a replica of the original dataset provides the privacy guarantee. Thus, the input of the mining task would be the synthetic dataset $X^{\prime }$.

### 3.4. Knowledge Distillation

Knowledge Distillation [18] allows building Machine Learning Copies that replicate the behavior of the learned decisions (e.g., Decision Trees rules) in the absence of sensible attributes. The idea behind the Knowledge Distillation is the compression of an already trained model. The technique generates a function updating parameters of a specific population to a smaller model without observing the training dataset’s sensitive variables. The methodology trains a binary classification model. Subsequently, the synthetic dataset is generated using different sampling strategies for the numerical and categorical attributes, maintaining the relationship between the independent variables and the dependent variable. Thus, new values are obtained for the variables in a balanced data group. Finally, the lower-dimensional synthetic dataset is used to train a new classification task with the same architecture and training protocol as the original model. The idea behind this algorithm is to create synthetic data for forming a new private aware dataset. Hence, we build a new dataset from a sampling process using uniform or normal distributions. The samples are validated by a classifier trained with the original dataset *X*. This technique allows building a dataset representation in another space, which becomes our sanitized dataset $X^{\prime }$.

### 3.5. Evaluation Metrics for Privacy Filters

To assess the quality of the sanitation algorithms in terms of information utility and privacy risk, we use two standard metrics in the literature, namely Information Loss and Disclosure Risk [1,2,3,4,5,6]. In the following paragraphs, we define how both functions are implemented.

Information Loss (IL)

Information Loss is a metric that quantifies the impact of a sanitization method on the dataset utility. It quantifies the amount of useful information lost after applying a sanitization algorithm, and there are several methods to compute it. In the present paper, we rely on the Cosine similarity measure between the original value of the salinity, chlorophyll, temperature, and degrees under the sea *X* and the vector $X^{\prime }$, which is the sanitized counterpart of *X* as defined in Equation (6).

(6) $$cosd\left(X,X^{\prime }\right)=1-\frac{X\cdot X^{\prime }}{{\left||X|\right|}_{2}*\left|\right|X^{\prime }{\left|\right|}_{2}}$$

Thus, to compute the IL, we sum the distances between the original *X* and sanitized $X^{\prime }$ vector of points using Equation (7).

(7) $$IL=\sum_{i=1}^{n}\left(cosd\left(X,X^{\prime }\right)\right)$$

Disclosure Risk (DR)

Disclosure risk quantifies the danger of finding the same distribution for the output variable after a prediction task when the input dataset is sanitized. For the sake of example, let *X* be the original dataset, containing salinity, chlorophyll, temperature, and degrees under the sea, and $X^{\prime }$ the sanitized version of *X*. Both datasets are the input of a Logistic Regression to predict the volume of fish stocks. Thus, the model outputs the prediction *Y* using the original dataset and $Y^{\prime }$ for the sanitized input.

Therefore, we use the Jensen-Shannon distance metric to measure the closeness between two vectors *Y* and $Y^{\prime }$. Where m is the average point of *Y* and $Y^{\prime }$ vectors, and *D* is the Kullback-Leibler divergence.

(8) $$DR=1-\sqrt{\frac{D(Y||m)+D(Y^{\prime }\left||m\right)}{2}}$$

In the experiments *Y* and $Y^{\prime }$ are the predicted vectors of a given model on the real and sanitized data, respectively.

Based on the aforementioned concepts, we performed some experiments whose results are reported in the next section.

## 4. Results

Inspired on a benchmark previously described in [35], we compare four groups of sanitization techniques: Statistical Disclosure Control filters, Differential Privacy filters, Generative Adversarial Networks, and Knowledge Distillation technique (The implementation of the privacy algorithms is available at: https://github.com/bitmapup/privacyAlgorithms accessed on 4 April 2021). These methods are applied to the dataset described below.

### 4.1. Dataset Description

We live in an interconnected world where much data is generated from sensors, social networks, internet activity, etc. Therefore many companies have important datasets, which are both economic and scientific valuables. Thus, it is necessary to analyze and understand sanitation techniques for curating commercial datasets to be shared publicly with the scientific community owing to their informative value. In this sense, we take the case of the fishing industry in Peru, which is one of the most important economic activities [36] for the Gross Domestic Product (GDP). In this economic activity, the cartographic charts are a high economic investment to understand where the fish stocks are located in the sea for maximizing the daily ship’s fishing. Simultaneously, this information is helpful to predict *el Niño* phenomenon and study the fish ecosystem.

**Figure 1:**
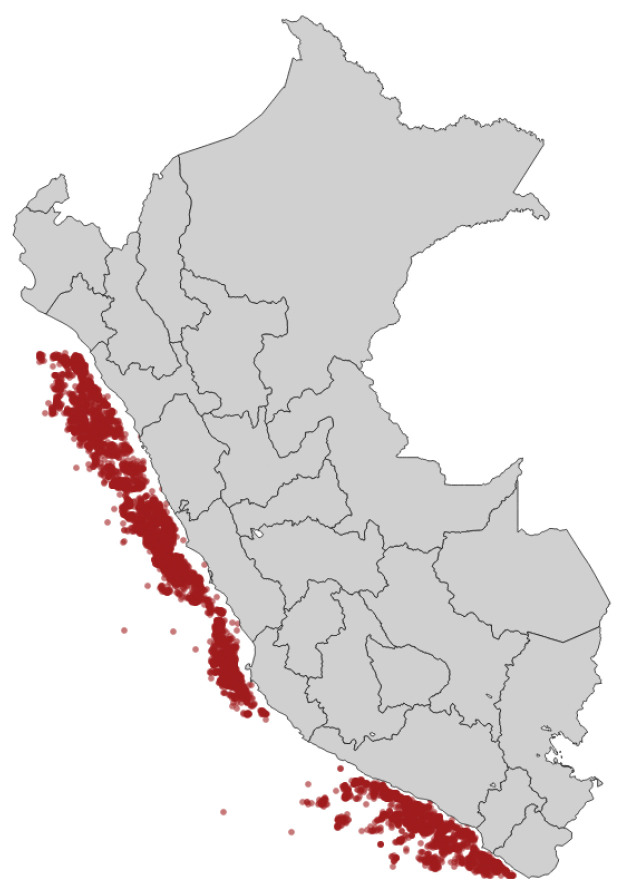
Spatial representation of dataset.

The oceanographic charts provide geo-referenced water characteristics data on the Peruvian coast as depicted in Figure 1. The overall dataset contains 9529 temporal-stamped records and 29 features, which are detailed in Table 1.

**Figure 2:**
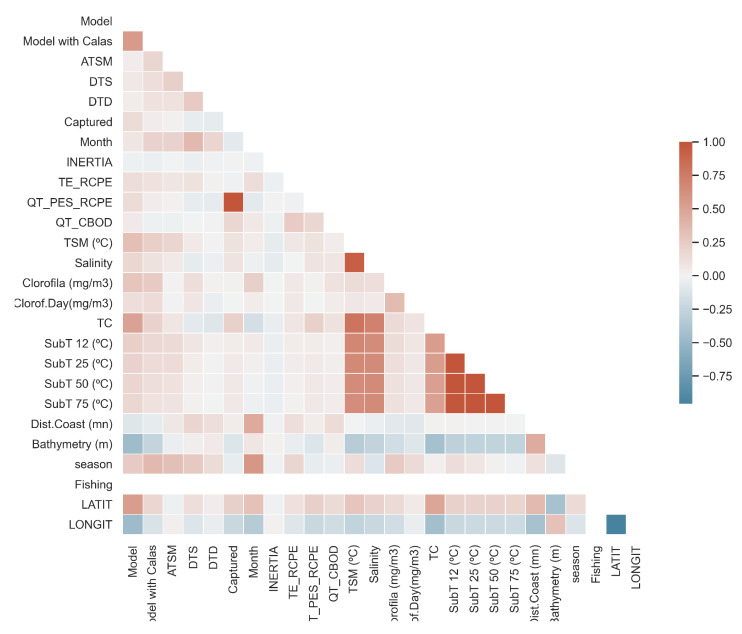
Feature correlation heatmap for the raw dataset.

From the variables before presented, the variables ranging from 19 to 22, in Table 1 were discarded due to the high correlation to degrees under the sea *TC* as depicted in Figure 2. Then, variables 1, 2, and 9 to 13 are not take into account because they belong to a in-house model. Another variable highly correlationated with *Chlorophyll* is *Chlorophyll per Day* (*Clorof.Day*) as shown in Figure 2. Finally, *Dist.Coast, Bathymetry, North-South* and *Season* have a poor predictive power for the mining task.

Therefore, four main characteristics are used for finding fish stock’s location. These features are *salinity, chlorophyll, temperature* (TSM), and *degrees under the sea* (TC), which are described in Table 2 (Dataset available at: https://dataverse.harvard.edu/dataset.xhtml?persistentId=doi:10.7910/DVN/IFZRTK accessed on 4 April 2021). Thus, in the present work, we limit the study to these four features used to find the fish stocks.

### 4.2. Data Sanitization through Statistical Disclosure Control Filters

This subsection shows the sanitization process using the Statistical Disclosure Control (SDC) filters. The SDC filters are applied using different settings to find the most suitable configuration (c.f., Table 3) for a good trade-off between Information Loss and Disclosure Risk metrics. Thus, we use different parameter settings to minimize privacy risks and maximize data utility.

Noise Addition

**Figure 3:**
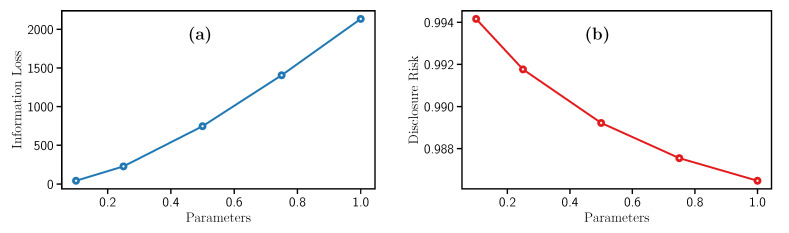
Evolution of the (**a**) Information Loss and the (**b**) Disclosure Risk with Noise addition filter.

This filter needs the parameter a=1, σ is the standard deviation of the variable, and *c*, which is a scaling factor for adding noise to each row in a dataset. In this experiments, *c* take values of 0.1, 0.25, 0.5, 0.75, and 1. Therefore, Figure 3a illustrates the Information Loss increment while *c* grows. Analogously, Figure 3b indicates that the Disclosure Risk follows a different behavior since it decreases while *c* increases. This monotonic decrease makes it more difficult to obtain the original data from the sanitized dataset.

In conclusion, high values of *c* represent strong privacy guarantees and data utility loss. Besides, this filter requires low computational time to process the data.

Microaggregation

This filter uses the spatial density-based DBSCAN [37] clustering algorithm. After the clustering step, each point in the dataset belonging to a cluster is replaced by the cluster’s mean value to sanitize it. Accordingly, DBSCAN uses the number of kilometers km each cluster will encompass and the minimum number of geospatial points m belonging to a cluster. In this effort, the km value was empirically set to 1, 2, 3, and 4; while m was set to 50, 100, 150, 200, 250, and 300. Both parameters were tested in all possible combinations to obtain the best results, as depicted in Table 4. It is worth noting that the number of formed clusters directly depends on both hyperparameters.

**Figure 4:**
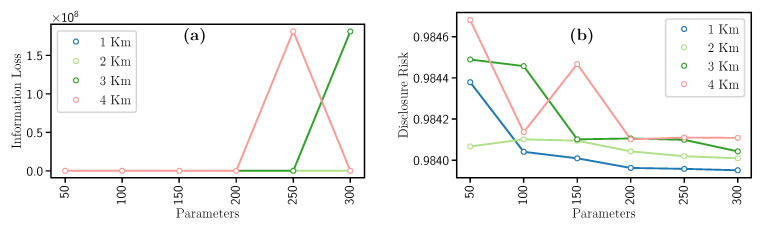
Evolution of the (**a**) Information Loss and the (**b**) Disclosure Risk with the Microagreggation filter.

Concerning the results, when variables km and m increase, values of Information Loss and Disclosure Risk have opposite behaviors, i.e., the Information Loss increases (see Figure 4a) and the Disclosure Risk decreases (see Figure 4b). In detail, we notice in Figure 4a, the higher values of km and m, the higher the loss of data utility since there are few clusters. Then, the more the clusters, the less Information Loss value. Furthermore, in the case of Disclosure Risk (Figure 4b), by increasing the value of km, the Disclosure Risk decreases since there are few clusters. Consequently, if km remains fixed and m increases, the Disclosure Risk decreases.

In general terms, as the value of km increases, the IL increases, and DR decreases. Also, while m increases, there is a greater guarantee of information privacy, and the loss of utility also decreases. This filter has a disadvantage due to the high computational time required.

Rank Swapping

This filter takes as input the maximum exchange range *p*. The experiments have been performed for *p* values varying from 10 to 80 (c.f. Table 3).

**Figure 5:**
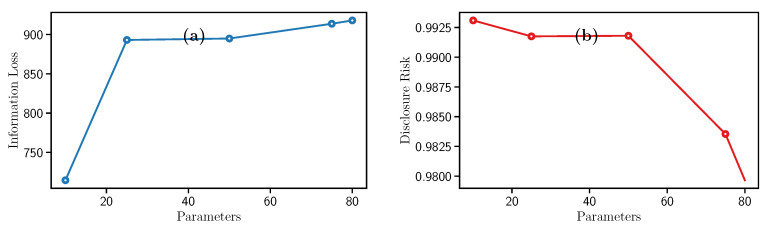
Evolution of the (**a**) Information Loss and the (**b**) Disclosure Risk with the Rank Swapping filter.

Concerning the results, Figure 5 shows that IL remains stable for *p* values from 25 to 80. On the opposite, concerning the DR the highest and lowest results are obtained for p=10 and p=80, respectively. It means that when *p* increases, there is less Disclosure Risk, making it more challenging to obtain the original data from the sanitized version.

To summarize, Figure 5a,b display that the Disclosure Risk has its lowest point when p=80. It means that we protect the data better despite taking away its usefulness. On the other hand, if you want to have the least amount of loss of the information usefulness, the hyperparameter p=10 is the best option. However, this filter is the one that requires the most computational time to sanitize the data and offers a better Disclosure Risk compared to the other SDC filters.

### 4.3. Data Sanitization through Differential Privacy Filters

In this section, two techniques based on Differential Privacy mechanisms were applied to data at our disposal. Experiments were performed in three parts. First, the Microaggregation filter was applied for different values of km and m. Once the clusters were obtained, the data was replaced by the mean value. Finally, Exponential and Laplacian Differential Privacy mechanisms were applied, each one with hyperparameters described in Table 5.

Laplacian Mechanism

**Figure 6:**
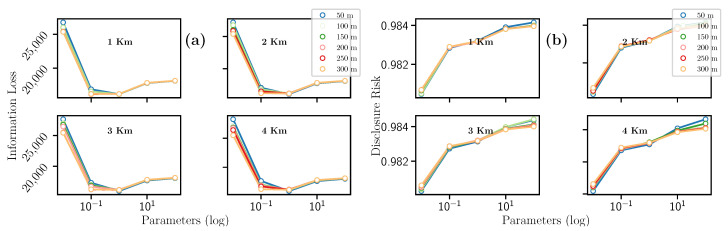
Evolution of the (**a**) Information Loss and the (**b**) Disclosure Risk with the Laplacian mechanism.

Additionally to km and m variables, the Laplacian mechanism uses ϵ that was set to 0.01, 0.1, 1, 10, and 100. The result for this filter can be summarized as follows. On the one hand, Figure 6a evinces that the hyperparameters km and m seem not to impact the Information Loss value. However, this metric decreases drastically when the hyperparameter ϵ=1. We also see that the Information Loss progressively grows, reaching a maximum peak when ϵ=0.01. This trend being fulfilled for all combinations of km and m.

On the other hand, Figure 6b indicates that the Disclosure Risk decreases when m increases. Analogously, as the km value increases, the Disclosure Risk also increases. Concerning the ϵ hyperparameter, there is a similar trend to the Information Loss metric, i.e., where the Disclosure Risk metric reaches its minimum point when ϵ=1, for constant values of km and m.

**Figure 7:**
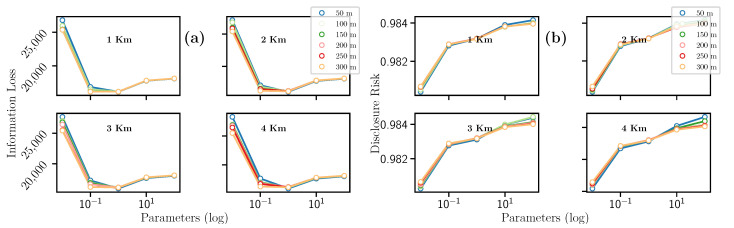
Evolution of the (**a**) Information Loss and the (**b**) Disclosure Risk with the Exponential mechanism.

To summarize, concerning the Information Loss (see Figure 7a), a quadratic trend is observed. The highest and lowest Information Loss peaks were obtained for km = 4 and m = 50, respectively. In the case of Disclosure Risk (see Figure 7b), a quadratic trend is also observed where the minimum point of Disclosure risk is given for ϵ=10. The maximum value of Disclosure Risk is obtained when km = 1, m = 100 and ϵ=0.01, and the minimum value when km = 1, m = 300 and ϵ=10. In conclusion, for constant values of km and m, only the value of ϵ allows us to guarantee high privacy when the value of this hyperparameter is equal to 0.01, or low when it is equal to 10. Please note that all values for IL and DR are summarized in Table 6.

Exponential Mechanism

As the Laplacian mechanism, the Exponential mechanism takes three hyperparameters: km, m, and ϵ. Regarding the Information Loss (c.f., Figure 7a), km and m seem to have no impact significant on this metric. Conversely, the Information Loss reaches a maximum peak for ϵ=0.01 and a minimum value when ϵ=1. Regarding the Disclosure Risk, Figure 7b shows that for km and m, there is a similar behavior described in the previous section. We can also notice that Disclosure Risk has the highest peak when the ϵ=0.01.

Regarding the Information Loss (c.f., Figure 7a), a quadratic trend is also observed. From ϵ>1 the IL starts to grow, reaching a maximum point when the hyperparameter ϵ=0.01. Similarly, the highest and lowest IL peaks are obtained when km = 4 and m = 50, respectively. It is important to notice that the IL value only depends on ϵ to obtain maximum or minimum values.

The Disclosure Risk (c.f., Figure 7b) also reveals a quadratic trend, where the minimum DR is at ϵ=10. Furthermore, the same trend can be observed in the km and m hyperparameters as in previous mechanism. The maximum value of all combinations is given when km = 1, m = 100 and ϵ=0.01, and the minimum DR when km = 1, m = 300 and ϵ=10. Please note that all values for IL (c.f., *Il exp*) and DR are summarized in Table 7.

### 4.4. Data Sanitization through Generative Adversarial Networks

In this section, a Generative Adversary Networks GAN is applied to data at our disposal, and the algorithm returns a dataset artificially generated through an Artificial Neural Networks (ANN) mechanism. Obtained results were evaluated by measuring the Disclosure Risk and the Information Loss.

During the training phase, the synthetic data generator *G* and the discriminator *D* models need parametrization. Different hyperparameters values generate completely different models with different results. Thus, we took the settings recommended in [38,39], which are summarized in Table 8. Concerning the number of hidden layers in the architecture of the ANN [38,39] recommends three hidden layers for each neural network (discriminator and generator). Also, the authors propose using the RELU activation function and Adam’s Optimizer fixed to 0.0001. Concerning the epochs, we were inspired by [38], which obtain good results using 300 and 500 epochs. Both epochs were empirically tested, obtained better results for 500 epochs.

In the same spirit, authors in [40] recommend training a GAN using the Batch technique, where the dataset is divided into *n* blocks of data and trained separately. This technique reduces training time. In addition, the recommended parameter *n* in the literature is 64. Finally, in [39,41], the authors use 100 neurons, and 100 input dimensions.

**Figure 8:**
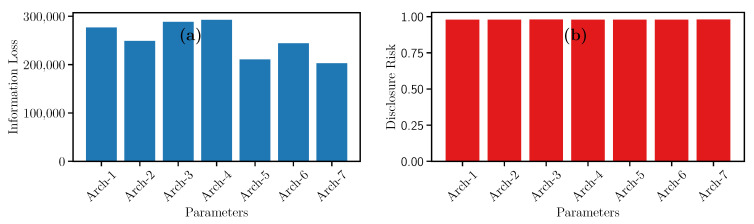
Evolution of (**a**) Information Loss and (**b**) Disclosure Risk measures for GAN architectures.

Concerning the result, in the case of the Information Loss (see Figure 8a), the highest peaks of the utility are found using Architecture 3 and Architecture 4. In contrast, Architecture 7 has the lowest Information Loss. Also, architectures 3 and 7 have the same number of hidden layers with 256, 512, and 2024 neurons. Nevertheless, both architectures have a significant difference concerning these values are positioned in the GAN. This difference generates a significant impact on IL.

### 4.5. Data Sanitization through Knowledge Distillation

To generate a synthetic dataset using Knowledge Distillation, we rely on Machine Learning copies. To meet this aim, the CART Decision Tree algorithm [45] was trained with the original normalized data using Entropy and Gini to measure the split’s quality. For the maximum depth of the tree, we tested values ranging from 2 to 50. Then, for the minimum number of samples required to split an internal node, we try the following values 0.01, 0.05, 0.1, 0.15, 0.16, 0.18, and 0.2. Table 9 summarizes the best values found for both Entropy and Gini based Decision Trees.

**Figure 9:**
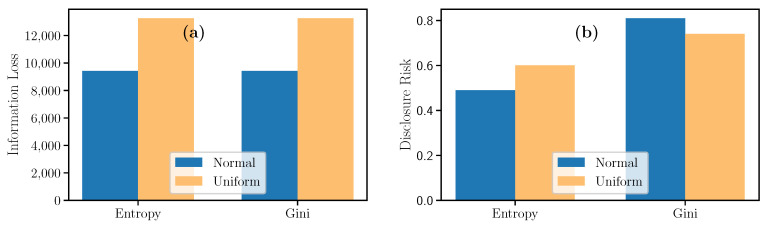
Evolution of Information Loss and Disclosure Risk measures for Knowledge Distillation.

Once the model is trained to outcome the pretense or absence of fish stocks given certain salinity values, chlorophyll, temperature, and degrees under the sea, a synthetic dataset was generates using random values sampled from normal and uniform distributions with parameters specified in Table 10. The obtained synthetic datasets were evaluated using the IL and the DR metrics. Figure 9 depicts the datasets issued from the normal distribution have less Information Loss and a similar Disclosure Risk between 0.5 and 0.8.

In this section, we have presented the results of the different techniques to benchmark the results in terms of Information Loss and Disclosure Risk. In the next section, we discuss about our findings.

## 5. Discussion

A vast amount of data are generated and collected daily. These datasets contain sensitive information about individuals, which needs to be protected for public sharing or mining tasks to avoid privacy breaches. As a consecuence, data curators have to choose a suitable technique to guarantee a certain privacy level while keeping a good utility for the mining task after the sanitization. There are several privacy-enhancing mechanisms based on SDC, Differential privacy, GANs, or Knowledge Distillation to protect the data. Thus, there is a need to compare such methods for data sanitizations. Therefore, a question about the best algorithm to protect privacy raise. To try to answer this question, we extend the benchmarks [24,35] from a comparison of classical Statistical Disclosure Control, and Differential Privacy approaches with recent techniques as Generative Adversarial Networks and Knowledge Distillation using a commercial database.

About the SDC filters, the highest possible Information Loss was obtained with the Microaggregation filter and the lowest possible Information Loss with the Rank swapping filter. Besides, the highest Disclosure Risk value was obtained using Rank swapping and Noise Addition, while the lowest Disclosure Risk value was achieved through the Microaggregation filter.

Regarding Differential Privacy, the Laplacian and Exponential mechanisms differ slightly for both Disclosure Risk and Information Loss. Thus, when ϵ=0.01, and ϵ=0.1, we obtain the lowest DL and the highest IL respectively. Depending on the data sanitization’s primary purpose, it is recommended to alternate these ϵ values with constant values of km and m. Since both Exponential and Laplacian mechanisms present almost the same values, it is recommended to use the Laplacian mechanism since it takes the least computational time to execute. Concerning the ϵ choice, we suggest small values close to zero to avoid privacy breach, since ϵ could be seen as the probability of receiving the same outcome on two different datasets [30], which in our case are the original dataset *X* and its private counterpart $X^{\prime }$.

The GAN technique shows that Architecture 3 should be used when a high privacy guarantee is required with a shallow Disclosure Risk measure. However, to have the least utility loss, it is recommended to opt for Architecture 7 or Architecture 5, since they have the lowest Information Loss. To decrease Disclosure Risk, it is possible to couple the GAN with a Differential Privacy mechanism, as mentioned in [15,16,17].

Concerning the Knowledge Distillation technique, despite the fact that the distillation process could change the class balance depending on the sampling strategy. It seems to shows interesting results in terms of Information Loss and Disclosure Risk. It is worth noting that the sampling process could be challenging depending on how the process sampling is done [46].

To summarize, Table 11 indicates the best trade-offs between Information Loss and Disclosure Risk measures for the compared methods. We observe that Machine Learning copies present the best trade-off between Information Loss and Disclosure Risk. Then, GAN provides the second-best privacy guarantee. The strategy of this method is different from classical SDC filters and differential privacy. The former algorithms build a dataset to mimic original counterparts, while the latter algorithms add controlled noise to the original data. Hence, We notice that Noise Addition and Rank Swapping have the smallest Information Loss values. Finally, we remark that Microagreggation and Differential Privacy have similar behaviors. Based on the results mentioned above, a data curator should first try a Machine Learning Copy to reduce privacy risk while keeping a small Information Loss when performing a mining task. The second option would be to try Differential Privacy after the Machine Learning Copy since it is the second-best trade-off.

Apropos computational time, the fastest sanitization algorithm, using our dataset, is Noise Addition, it takes on average 30 min to execute. Then, Rank Swapping, Microaggregation, Differential Privacy, and GANs take about 2 h to execute, and Machine Learning Copies could take more than two hours depending on the sampling strategy and previous knowledge on the probability distributions of the variables corresponding to the dataset to be sanitized.

It is worth noting that latitude and longitude variables were not considered in the sanitization process since SDC methods change in an arbitrary way when treated as variables. This could degrade the dataset significantly while working with geo-referenced data. Thus, an adversary could note that the dataset has been previously processed. The risk of dealing with geolocation data are detailed in [47]. Also, to the best of our knowledge, there are no studies about the privacy preservation of geolocated records using GANs or Machine Learning copies. Concerning IL and DR, there is not a consensus about the definition of such a function. Thus, there is an opportunity to implement different functions to capture the impact of the privacy mechanism. Besides, it is possible to extend this study by testing the used sanitization techniques with other datasets, such as medical datasets like the one presented in [48]. The limitation is that authors do not share the analyzed dataset and, in general, the unavailability of public available medical datasets. Another angle of analysis is the subsequent mining task after the sanitization. Consequently, one can test different data mining tasks, namely, classification, clustering, or sequential pattern mining, to evaluate the sanitization method’s impact on the result of the mining task and the information loss.

Concerning the context of our work, we have seen in the literature benchmarks of de-identification techniques [21,22] limited to record anonymity techniques, which are the first step of the sanitization process, on the one hand. On the other hand, other benchmarks compare SDC and Differential Privacy techniques [23,24,25,35] excluding deep learning-based approaches. To the best of our knowledge, this benchmark is the first one to compare classical SDC and Differential Privacy methods to Generative Adversarial Networks and Knowledge Distillation based privacy techniques. Therefore, this benchmark could be the first reference to guide data curators to choose a suitable algorithm for their sanitization task. Regarding the limitations of our work, even though the limited number of datasets used for the experiments, the results are quite convincing about the privacy gain by reducing the disclosure risk with a controlled information loss depending on the hyperparameters. Besides, our results are similar to those presented in [24,25,35], which took into account different datasets for their experiments. In conclusion, we have developed an extensive comparison of different privacy techniques regarding Information Loss and Disclosure Risk to guide in choosing a suitable strategy for data sanitation. There are several privacy techniques to sanitize datasets for public sharing. Thus, our contribution aims to fill the absence of privacy algorithms benchmark for proving the first approach to find a suitable sanitization technique. Therefore, our study could help to reduce the amount of time for selecting a privacy algorithm for data sanitization.

Because of this paper’s results, we are now able to evaluate GANS and Machine Learning copies for handling geolocated data and assess the impact of the privacy techniques when dealing with location data and other variables.

## 6. Conclusions

In the present effort, we have evaluated SDC (Statistical Disclosure Control) filters, namely Noise Addition, Microaggregation, Rank swapping, Laplacian and Exponential Differential Privacy, Generative Adversarial Networks (GAN), and Knowledge Distillation sanitization techniques on data using oceanographic charts. Therefore, the idea was to use the sanitized dataset for a fish stock prediction task. To calibrate the sanitization algorithms, different settings were tested for each technique. Thus, we evaluate the sanitization techniques in terms of Information Loss and Disclosure Risk. In this way, the best hyperparameter configurations were found, which achieve a trade-off between the Information Loss and the Disclosure Risk for each filter studied in this paper. However, there is room for improvements in testing the different techniques on other datasets and monitoring the computational time and memory usage using different hyperparameter values. This benchmark could be a good start for a data curator to target the most suitable privacy algorithm better to sanitize their datasets. Finally, the new research avenues will be to perform the benchmark by using publicly available datasets, monitor computational performance indicators like computational time and memory usage for all the filters with different configurations to analyze the hyperparameters’ impact on performance. Other experiments would be adding records’ geolocation and coupling Differential Privacy to the GANs and the Machine Learning Copies. However, there is room for improvements in testing the different techniques on other datasets and monitoring the computational time and memory usage using different hyperparameter values.

## Figures and Tables

**Table 1:** List of variables of the raw dataset.

Num.	Variable	Description
1	Model	Results of the company in-house model
2	Model with Calas	Results of the company in-house model
3	ATSM	A subtype of Temperature (TSM)
4	DTS	Variable for the in-house model
5	DTD	Variable for the in-house model
6	Hour	Hour when the data was obtained
7	Captured	Number of tons fished
8	Month	The month when the data was obtained
9	INERTIA	Variable for the in-house model
10	CO_RCPE	Variable for the in-house model
11	TE_RCPE	Variable for the in-house model
12	QT_PES_RCPE	Variable for the in-house model
13	QT_CBOD	Variable for the in-house model
14	TSM (ºC)	Temperature in °C
15	Salinity	Salinity
16	Chlorophyll (mg/m${}^{3}$)	The chlorophyll of the water in milligrams by cubic meter
17	Chlorop.Day(mg/m${}^{3}$)	The chlorophyll of the water with adjustments
18	TC	Degrees centigrade underwater
19	SubT 12 (ºC)	Degrees centigrade 12 m underwater
20	SubT 25 (ºC)	Degrees centigrade 25 m underwater
21	SubT 50 (ºC)	Degrees centigrade 50 m underwater
22	SubT 75 (ºC)	Degrees centigrade 75 m underwater
23	Dist.Coast (mn)	Distance from the beach
24	Bathymetry (m)	Bathymetry expressed in meters
25	North-South	North or south where data was obtained
26	Season	Semester of the year where data was obtained
27	Fishing	Do we found fish? (Yes = 1, No = 0)
28	LATIT	Latitude where data was collected
29	LONGIT	Longitude where data was obtained

**Table 2:** Characteristics of the dataset.

Features	Minimum Range	Maximum Range
Salinity	32.99	35.61
Chlorophyll	0.01	64.565
Temperature (TSM)	15	26
Degrees under the sea (TC)	26	150

**Table 3:** Values of hyperparameters.

Filter	Hyperparameter	Values
Noise addition	c	0.1, 0.25, 0.5, 0.75, 1.0
Microagregation	km	1, 2, 3, 4
	m	50, 100, 150, 200, 250, 300
Rank swapping	p	10, 25, 50, 75, 80

**Table 4:** Microaggregation results in Figure 4.

Km	m	IL	DR	Km	m	IL	DR
1	50	18,174.30	0.984379	3	50	18,165.75	0.984489
	100	18,173.20	0.984040		100	18,163.71	0.984457
	150	18,172.10	0.984300		150	18,159.74	0.984101
	200	18,169.94	0.983962		200	18,153.91	0.984105
	250	18,165.78	0.983957		250	18,141.69	0.984098
	300	18,152.95	0.983950		300	180,955,821	0.984042
2	50	18,169.94	0.984066	4	50	18,161.69	0.984681
	100	18,167.86	0.984101		100	18,158.77	0.984136
	150	18,165.75	0.984094		150	18,153.91	0.984466
	200	18,161.71	0.984042		200	18,145.97	0.984101
	250	18,153.91	0.984019		250	181,287,912	0.984101
	300	18,126.88	0.984009		300	18,063.54	0.984108

**Table 5:** Values of hyperparameters.

Privacy Filter	Parameter	Value
Microagreggation-Laplacian Mechanism	km	1, 2, 3, 4
	m	50, 100, 150, 200, 250, 300
	ϵ	0.01, 0.1, 1, 10, 100
Microagreggation-Exponential Mechanism	km	1, 2, 3, 4
	m	50, 100, 150, 200, 250, 300
	ϵ	0.01, 0.1, 1, 10, 100

**Table 6:** IL and DR values for different Laplacian Differential Privacy settings used in Figure 6.

Km	m	ϵ	IL exp	DR exp	Km	m	ϵ	IL exp	DR exp
		0.01	26,756.39	0.9805			0.01	27,652.85	0.980369
		0.1	16,865.94	0.98287			0.1	17,303.98	0.982801
1	50	1	16,149.69	0.983187	3	50	1	15,960.96	0.983196
		10	17,786.03	0.983802			10	17,673.83	0.983982
		100	18,112.68	0.984001			100	18,048.13	0.984428
		0.01	26,013.5	0.98065			0.01	26,990.42	0.980451
		0.1	16,547.53	0.982883			0.1	16,961.78	0.982813
1	100	1	16,164.33	0.983177	3	100	1	16,114.65	0.983194
		10	17,810.92	0.98382			10	17,762.22	0.983883
		100	18,127.01	0.983965			100	18,099.91	0.984125
		0.01	25,402.85	0.98068			0.01	26,775.29	0.980495
		0.1	16,197.18	0.982872			0.1	16,861.32	0.982845
1	150	1	16,155.76	0.983161	3	150	1	16,157.64	0.98321
		10	17,817.23	0.983815			10	17,787.94	0.983862
		100	18,131.56	0.983958			100	18,113.86	0.984086
		0.01	25,394.53	0.980687			0.01	26,462.76	0.980591
		0.1	16,186.31	0.982899			0.1	16,701.03	0.982874
1	200	1	16,177.68	0.983185	3	200	1	16,166.38	0.983171
		10	17,820.39	0.983815			10	17,796.87	0.983838
		100	18,133.91	0.983956			100	18,120.25	0.984028
		0.01	25,376.96	0.980685			0.01	25,475.35	0.980605
		0.1	16,153.66	0.982899			0.1	16,223.53	0.982872
1	250	1	16,177.4	0.983183	3	250	1	16,136.36	0.98318
		10	17,822.5	0.983809			10	17,804.01	0.983837
		100	18,135.17	0.983953			100	18,124.43	0.984006
		0.01	25,354.64	0.980468			0.01	25,452.33	0.980274
		0.1	16,172.67	0.982834			0.1	16,235.95	0.982729
1	300	1	16,173.75	0.983205	3	300	1	16,142.72	0.983137
		10	17,825.67	0.983904			10	17,808.02	0.983982
		100	18,136.47	0.98415			100	18,126.79	0.984383
		0.01	27,183.42	0.980452			0.01	28,277.14	0.980372
		0.1	17,055.7	0.982844			0.1	17,643.9	0.982784
2	50	1	16,061.64	0.983183	4	50	1	15,906.35	0.983196
		10	17,732.91	0.983891			10	17,613.26	0.983939
		100	18,083.27	0.984126			100	18,013.36	0.984153
		0.01	26,747.83	0.980516			0.01	27,172.31	0.980408
		0.1	16,822.59	0.982869			0.1	17,063.76	0.982813
2	100	1	16,160.54	0.983171	4	100	1	16,068.98	0.983221
		10	17,789.16	0.983822			10	17,733.87	0.983949
		100	18,113.77	0.98406			100	18,084.69	0.98439
		0.01	26,020.64	0.980629			0.01	26,878.98	0.980443
		0.1	16,519.16	0.982864			0.1	16,929.86	0.982817
2	150	1	16,160.54	0.983175	4	150	1	16,125.12	0.98317
		10	17,801.84	0.983839			10	17,770.89	0.98389
		100	18,122.52	0.984007			100	18,104.74	0.984123
		0.01	25,465.32	0.980507			0.01	26,764.21	0.980493
		0.1	16,225.49	0.982884			0.1	16,874.04	0.982854
2	200	1	16,140.29	0.983209	4	200	1	16,149.25	0.98318
		10	17,807.84	0.983787			10	17,787.25	0.983869
		100	18,126.74	0.983978			100	18,113.82	0.984098
		0.01	25,864	0.980666			0.01	26,425.79	0.980616
		0.1	16,480.83	0.982871			0.1	16,697.03	0.982888
2	250	1	16,177.41	0.983176	4	250	1	16,163.15	0.983194
		10	17,814.85	0.983822			10	17,797.02	0.983849
		100	18,129.3	0.983972			100	18,119.35	0.984065
		0.01	25,384.07	0.980334			0.01	25,512.36	0.980179
		0.1	16,199.82	0.982801			0.1	16,219.92	0.982717
2	300	1	16,164.44	0.983173	4	300	1	16,126.39	0.983086
		10	17,815.22	0.983932			10	17,800.4	0.984091
		100	18,131.55	0.984151			100	18,122.4	0.984654

**Table 7:** IL and DR values for different Exponential Differential Privacy settings used in Figure 7.

Km	m	ϵ	IL exp	DR exp	Km	m	ϵ	IL exp	DR exp
		0.01	26,805.88	0.980468			0.01	27,655.44	0.980274
		0.1	16,874.44	0.982834			0.1	17,297.23	0.982729
1	50	1	16,147.63	0.983205	3	50	1	15,968.72	0.983137
		10	17,786.58	0.983904			10	17,671.17	0.983982
		100	18,112.87	0.98415			100	18,047.94	0.984383
		0.01	25,963.42	0.9805			0.01	27,002.51	0.980369
		0.1	16,526.59	0.98287			0.1	16,956.95	0.982801
1	100	1	16,179	0.983187	3	100	1	16,116.49	0.983196
		10	17,810.1	0.983802			10	17,762.91	0.983982
		100	18,126.96	0.984001			100	18,099.91	0.984428
		0.01	25,414.27	0.98065			0.01	26,773.04	0.980451
		0.1	16,203.65	0.982883			0.1	16,864.37	0.982813
1	150	1	16,164.88	0.983177	3	150	1	16,155.21	0.983194
		10	17,815.58	0.98382			10	17,787.78	0.983883
		100	18,131.49	0.983965			100	18,113.81	0.984125
		0.01	25,364.12	0.98068			0.01	26,416.96	0.980495
		0.1	16,170.88	0.982872			0.1	16,674.77	0.982845
1	200	1	16,166.61	0.983161	3	200	1	16,166.32	0.98321
		10	17,820.4	0.983815			10	17,799.32	0.983862
		100	18,133.81	0.983958			100	18,120.24	0.984086
		0.01	25,372.59	0.980687			0.01	25,480.51	0.980591
		0.1	16,176.96	0.982899			0.1	16,228.98	0.982874
1	250	1	16,168.93	0.983185	3	250	1	16,132.41	0.983171
		10	17,822.96	0.983815			10	17,803.37	0.983838
		100	18,135.04	0.983956			100	18,124.54	0.984028
		0.01	25,363.77	0.980685			0.01	25,416.3	0.980605
		0.1	16,171.44	0.982899			0.1	16,210.68	0.982872
1	300	1	16,173.73	0.983183	3	300	1	16,145.41	0.98318
		10	17,822.86	0.983809			10	17,807.97	0.983837
		100	18,136.35	0.983953			100	18,126.75	0.984006
		0.01	27,155.12	0.980334			0.01	28,302.08	0.980179
		0.1	17,068.17	0.982801			0.1	17,644.73	0.982717
2	50	1	16,055.25	0.983173	4	50	1	15,897.25	0.983086
		10	17,732.17	0.983932			10	17,617.03	0.984091
		100	18,083.42	0.984151			100	18,013.25	0.984654
		0.01	26,821.81	0.980452			0.01	27,147.58	0.980372
		0.1	16,841.09	0.982844			0.1	17,043.56	0.982784
2	100	1	16,160.23	0.983183	4	100	1	16,061.7	0.983196
		10	17,787.89	0.983891			10	17,734.34	0.983939
		100	18,113.78	0.984126			100	18,084.73	0.984153
		0.01	25,989	0.980516			0.01	26,906.04	0.980408
		0.1	16,539.61	0.982869			0.1	16,907.99	0.982813
2	150	1	16,152.18	0.983171	4	150	1	16,127.52	0.983221
		10	17,804.2	0.983822			10	17,771.35	0.983949
		100	18,122.63	0.98406			100	18,104.71	0.98439
		0.01	25,444.78	0.980629			0.01	26,768.91	0.980443
		0.1	16,207.17	0.982864			0.1	16,825.51	0.982817
2	200	1	16,133.43	0.983175	4	200	1	16,165.04	0.98317
		10	17,807.95	0.983839			10	17,786.04	0.98389
		100	18,126.7	0.984007			100	18,113.89	0.984123
		0.01	25,865.46	0.980507			0.01	26,464	0.980493
		0.1	16,473.8	0.982884			0.1	16,702.41	0.982854
2	250	1	16,180.91	0.983209	4	250	1	16,152.97	0.98318
		10	17,814.25	0.983787			10	17,797.78	0.983869
		100	18,129.6	0.983978			100	18,119.23	0.984098
		0.01	25,403.73	0.980666			0.01	25,510.33	0.980616
		0.1	16,221.69	0.982871			0.1	16,238.83	0.982888
2	300	1	16,147.73	0.983176	4	300	1	16,121.84	0.983194
		10	17,817.59	0.983822			10	17,799.11	0.983849
		100	18,131.55	0.983972			100	18,122.33	0.984065

**Table 8:** Values for different architectures.

Privacy Filter	Parameters	Selected Values
	Architecture-1 [42]	*G*: 32-64-128 *D*: 128-64-32
	Architecture-2 [39]	*G*: 50-50-50 *D*: 50-50-50
	Architecture-3 [38]	*G*: 1024-512-256 *D*: 256-512-1024
GAN	Architecture-4 [39]	*G*: 256-512-1024 *D*: 512-512-512
	Architecture-5 [43]	*G*: 512-512-512 *D*: 512-512-512
	Architecture-6 [44]	*G*: 128-128-64 *D*: 64-128-256
	Architecture-7 [34]	*G*: 256-512-1024 *D*: 1024-512-256

**Table 9:** Decision tree parameters.

Criterion	Gini	Entropy
max depth	20	10
min sample split	0.05	0.01

**Table 10:** Parameters for the Normal and Uniform distributions for data sampling.

Distribution	Variable	Majority Class Mean	Majority Class Std dev.	Minority Class Lower Bound	Minority Class Upper Bound
Normal	Salinity	−0.040169	1.095034	0.105732	0.678991
Normal	Temperature	−0.096785	1.052992	0.251601	0.804940
Normal	Chlorophyll	−0.014185	0.980216	0.028919	1.028593
Normal	Degrees under the sea	−0.043937	1.081239	0.117393	0.728411
Uniform	Salinity	−5.220127	0.238858	−5.220127	0.240401
Uniform	Temperature	−3.677595	1.079318	−3.677595	1.079318
Uniform	Chlorophyll	0.618748	10.093664	−0.618748	10.470102
Uniform	Degrees under the sea	−4.851172	1.643433	−4.851172	1.616258

**Table 11:** Best trade-offs between IL and DR.

Filter	Value of IL	Value of DR
Noise addition	2134	0.994
Microagreggaction	18,152.95	0.983950
Rank swapping	917.83	0.9925
Differential privacy Laplacian	17,822.50	0.983809
Differential privacy Exponential	17,822.96	0.983815
GAN	20,298.3	0.980435
Machine Learning copies	9424.05	0.809793

## Data Availability

Dataset available at: https://dataverse.harvard.edu/dataset.xhtml?persistentId=doi:10.7910/DVN/IFZRTK, accessed on 4 April 2021.

## Abbreviations

The following abbreviations are used in this manuscript:

IL	Information Loss
DR	Disclosure Risk
SDC	Statistical Disclosure Control
GAN	Generative Adversarial Networks
GDP	Gross domestic product
